# Techniques for Penile Augmentation Surgery: A Systematic Review of Surgical Outcomes, Complications, and Quality of Life

**DOI:** 10.3390/medicina60050758

**Published:** 2024-05-02

**Authors:** Ugo Giovanni Falagario, Federico Piramide, Karl H. Pang, Emil Durukan, Lazaros Tzelves, Anna Ricapito, Loic Baekelandt, Enrico Checcucci, Diego M. Carrion, Carlo Bettocchi, Francesco Esperto

**Affiliations:** 1 Department of Molecular Medicine and Surgery, Karolinska Institutet, 17177 Stockholm, Sweden; ugofalagario@gmail.com; 2 Department of Urology and Kidney Transplantation, University of Foggia, 71122 Foggia, Italy; carlo.bettocchi@unifg.it; 3 Department of Oncology, Division of Urology, University of Turin, San Luigi Gonzaga Hospital, 10043 Turin, Italy; federico.piramide@gmail.com; 4 Academic Urology Unit, University of Sheffield, Sheffield S10 2TN, UK; karlpang@doctors.org.uk; 5 Department of Urology, Copenhagen University Hospital, Herlev and Gentofte Hospital, 2730 Herlev, Denmark; emildurukan@gmail.com; 6 Second Department of Urology, National and Kapodistrian University of Athens, Sismanogleio General Hospital, 11527 Athens, Greece; lazarostzelves@gmail.com; 7 Department of Urology, University Hospitals Leuven, 3000 Leuven, Belgium; loic.baekelandt@uzleuven.be; 8 Department of Surgery, FPO-IRCCS Candiolo Cancer Institute, 10060 Turin, Italy; checcu.e@hotmail.it; 9 Department of Urology, Torrejon University Hospital, 28850 Madrid, Spain; diegocarrionm@gmail.com; 10 Universidad Francisco de Vitoria, 28223 Madrid, Spain; 11 Department of Urology, Campus Biomedico University of Rome, 00128 Rome, Italy; francescoesperto@gmail.com

**Keywords:** penile augmentation, penile enlargement, surgical techniques

## Abstract

The increase in practices related to enhancing penile size can be attributed to the belief that an improved genital appearance contributes to a man’s virility, coupled with an altered self-perception of his body. It is crucial to tailor interventions to meet the genuine needs of patients by thoroughly assessing their history, psychological state, and potential surgical benefits, all while considering the associated risks of complications. This systematic review aims to summarize the available evidence on outcomes, complications, and quality of life after penile augmentation surgery, examining both minimally invasive and more radical techniques. A search of the PubMed and Scopus databases, focusing on English-language papers published in the last 15 years, was performed in December 2023. Papers discussing surgery in animal models and case reports were excluded from the present study unless further evaluated in a follow-up case series. The primary outcomes were changes in penile dimensions, specifically in terms of length and girth, as well as the incidence of surgical complications and the impact on quality of life. A total of 1670 articles were retrieved from the search and 46 were included for analysis. Procedures for penile length perceived enhancements include lipoplasty, skin reconstruction plasty, V-Y and Z plasty, flap reconstruction, scrotoplasty, ventral phalloplasty, and suspensory ligament release; techniques for increasing corporal penile length include penile disassembly, total phalloplasty, and sliding elongation. Finally, penile girth enhancement may be performed using soft tissue fillers, grafting procedures, biodegradable scaffolds, and Penuma^®^. In conclusion, while penile augmentation surgeries offer potential solutions for individuals concerned about genital size, the risks and complexities need to be accounted for.

## 1. Introduction

Penile augmentation surgery, also known as penile enhancement or penis enlargement surgery, is a surgical procedure that aims to increase the length and/or girth of the penis. The history of penile augmentation surgery dates to ancient civilizations, where the practice of enlarging the penis through various methods, such as stretching or tying weights to the penis, was reported. In recent years, the popularity of penile augmentation surgery has increased significantly [1]. The growing demand for penile augmentation surgeries is driven by various factors such as increasing awareness about the availability of these procedures in combination with the availability of different techniques. These procedures are considered to be highly controversial, and the associated risks and complications are significant and can lead to permanent erectile dysfunction, penile deformity, sensory loss, or infections [2,3,4].

Men’s sexual function and satisfaction are affected by their discomfort with genital size, which motivates them to seek out invasive and non-invasive penis augmentation options [5]. Therefore, an appropriate psychological evaluation is crucial in patients intending to undergo penile enhancement surgery [6]. Small penis anxiety (SPA) is a condition some men with normal-sized penises may experience, where they feel substantially distressed about the length of their penis [7]. Moreover, the condition can be classified as a body dysmorphic disorder if the patient experiences obsessive behaviors, significant psychological distress, and SPA present at least 1 h per day [4].

Penile augmentation surgeries are primarily performed in men with sexual dysfunction caused by anatomical abnormalities, such as Peyronie’s Disease, or in men with true congenital micropenis (stretched penile length of less than 2.5 SD below the mean for age or stage of sexual development) [8].

A variety of techniques have been developed for enhancing the length and girth of the penis, each with its own set of advantages and disadvantages. The results of invasive and non-invasive strategies remain uncertain [9], with most techniques being supported by only case-series reports [10,11].

The following review aims to compare surgical outcomes, quality of life, and complications of non-invasive and invasive approaches to penile augmentation surgery.

### Indication for Penile Augmentation Surgery

A complete clinical evaluation should always be performed before surgery, and it should include a detailed medical history, a psychiatric/psychosexual evaluation, and an accurate physical examination with measurement of penis diameters [12], biochemical/sex hormone serum profiles, and an ultrasound examination in the flaccid and erect penis.

For lengthening surgery, the measurements of the flaccid, stretched, and erect penis post pharmacological stimulation are essential to obtain a quantitative idea of the possible gain for each patient. The stretched penile length (SPL) represents the most overlapping measurement of the erect penis, corresponding to the distance between the pubic symphysis and the apex of the glans [13]. For enlargement surgery, the circumference measurements of the flaccid and erect penis at the distal third of the shaft, just below the glans, at the middle third, and at the proximal third at the level of the penopubic junction are important to evaluate a possible gain on girth. 

Before planning any treatment, it is important to understand if the patient’s penis size is within the normal range, which for a Caucasian man corresponds to a mean length of 9.16 (SD 1.57) cm for flaccid and 13.24 (SD 1.89) cm for a stretched penis, as well as an average circumference of 9.31 (SD 0.9) cm for flaccid and 11.66 (SD 1.1) cm for an erect penis [14].

Clinical evaluation and the preliminary psychiatric evaluation can help in discerning those patients who would benefit from medical therapy or minimally invasive treatments from those who would benefit from surgery [12,15].

## 2. Material and Methods

A systematic literature search of the PubMed, Web of Science, and Scopus databases was conducted in December 2023 to identify English-language papers on penile augmentation surgery published within the last 15 years. The systematic review was performed according to the PRISMA guidelines [16]. Research terms used for the research were the following: “((Penile augmentation surgery) OR (penile enhancement)) OR (penis enlargement surgery)”. Review articles, editorials, commentaries, and research letters were included only if deemed to contain pertinent information on penile augmentation surgery. Papers discussing surgery in animal models and case reports were excluded from the present study unless further evaluated in a follow-up case series. The primary outcomes were changes in penile dimensions, surgical complications, and quality of life. Data on flaccid, stretched, and/or erect penile length, as well as patient satisfaction and quality of life, were extracted when available. Results from individual studies were synthesized and presented in tables, indicating mean/median preoperative and postoperative differences in penile length and girth. Initial screening of titles and abstracts for potential inclusion was conducted independently by two authors (U.G.F. and A.R.), while full-text screening involved four reviewers (U.G.F., F.P., K.P. and E.D.). Reference assessments were also performed for inclusion. Disagreements in article selection were resolved among the four reviewers.

## 3. Results

**Figure 1:**
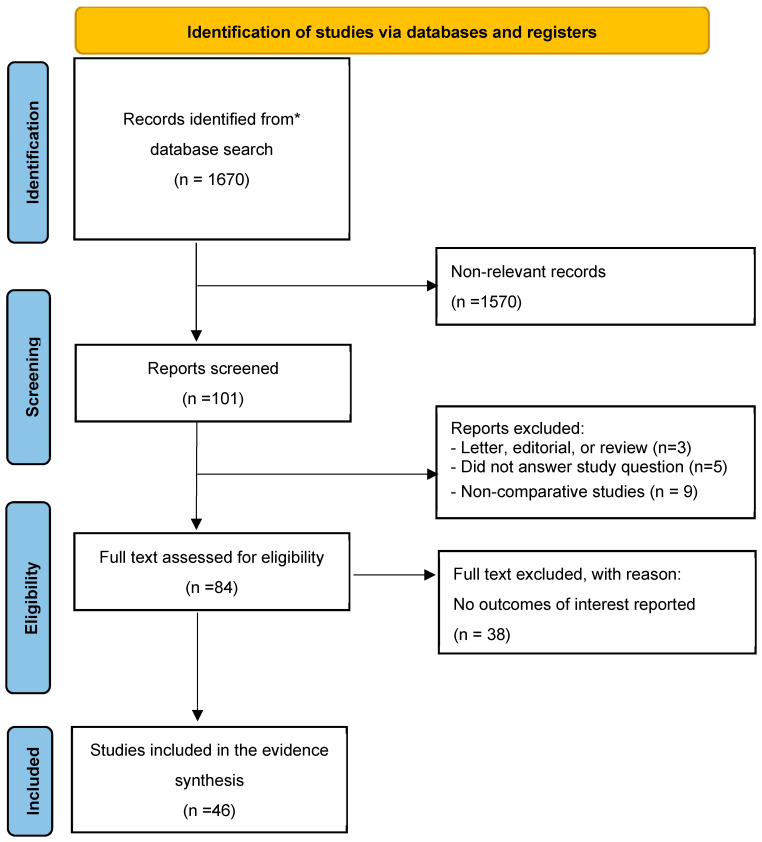
Preferred Reporting Items for Systematic Reviews and Meta-analysis (PRISMA) flow chart. * Search performed on PubMed.

Following the removal of duplicates, the search retrieved 1670 articles. A total of 101 articles were deemed relevant for screening. Eighty-four articles were included for full-text screening following the abstract screening stage, and a total of 46 articles were included for analysis (Figure 1).

Surgical interventions for enhancing penile size encompass methods aimed at increasing both length and girth. Furthermore, procedures for extending penile length can be categorized into those enhancing the perceived length and those increasing corporal length, whether or not penile prosthesis implantation is involved.

### 3.1. Techniques for Improving Perceived Penile Length

Usually, evaluation could be led by the presence or absence of a specific clinical picture, such as for acquired buried penis (ABP), which presents itself as a “false” micropenis. These patients should first undergo lipoplasty, and only in case of inadequate penis size should a further intervention be considered [17]. Until now a combination of multiple surgical approaches seemed to be the most suitable solution for surgeons and patients. The characteristics of studies on the techniques for increased penile length are summarized in Table 1.

#### 3.1.1. Lipoplasty

Lipoplasty is usually performed alone or in combination with another procedure to manage a buried penis secondary to obesity. This can be in the form of liposuction or panniculectomy [9,40]. A small, blunt cannula is usually used when performing suction lipectomy at the base of the penis. However, suction lipectomy alone is generally ineffective for the correction of a buried penis but can be successful in conjunction with panniculectomy.

In a panniculectomy, the suprapubic fat is excised down to the level of the abdominal fascia, and the dead space and skin are closed in layers. Patients who have a diseased shaft skin or retracted penis due to previous circumcisions may require further reconstruction in the form of a penile shaft skin (±foreskin) excision and a split-skin graft taken from the inner thigh [17,41]. Furthermore, if the buried penis is associated with a penoscrotal web, a scrotoplasty may be performed (see later). A urethral catheter is left in situ and the penile skin graft is dressed and/or vacuum-assisted for 10–14 days [17]. The graft take rate is over 80%, and the wound infection and dehiscence rate is around 10–20%. Overall, the patient satisfaction rate is between 80 and 90% [17].

#### 3.1.2. Skin Reconstruction Plasty

There are a variety of skin-related reconstruction procedures that help to increase penile length, such as V-Y and Z plasty, ventral phalloplasty, and scrotoplasty [9,42]. These procedures can also be used to lengthen the penis during penile curvature surgery for Peyronie’s disease [2] and inflatable penile prosthesis insertion [43].

### 3.2. V-Y and Z Plasty

The V-Y and Z plasty describes the shape of the incision and closure and can be performed on the penile shaft or foreskin, peno-scrotal, or penopubic junction. The aim is to increase the perceived length of the penis. These procedures can also be used to manage phimosis in patients who do not wish to undergo a full circumcision. In a Z plasty, triangles made at 60-degree angles when transposed can lead to a 1.75× increase in length [18].

### 3.3. Flap Reconstruction

Several flap reconstructions have been described [9]. The penopubic skin can be advanced onto the penile shaft by an inverted V-Y advancement flap. Other described skin flaps used to lengthen the penis include the lower abdominal Z plasty and the W-flap reconstruction [9].

Westerman et al. described a ventral slit scrotal flap (VSSF) as a new surgical option for buried penis syndrome which avoids complex skin grafting. This day case procedure involves an initial ventral slit made in the phimotic ring and exposing the penis. To cover the defect in the ventral shaft skin, local flaps are created by making a ventral midline scrotal incision with horizontal relaxing incisions. The scrotal flaps are rotated to resurface the ventral shaft. Fifteen consecutive patients with a penis buried due to lichen sclerosis or phimosis underwent repair with VSSF. At a mean follow-up of 12 months, 73.3% of men remain satisfied with their results and have required no further intervention. Recurrences occurred in 3 (20.0%) patients [44].

### 3.4. Ventral Phalloplasty or Scrotoplasty

A high insertion of the penoscrotal junction on the penile shaft skin may be inborn or acquired from excessive removal of the foreskin during circumcision. A ventral phalloplasty involves a vertical incision parallel to the phallus ~1 cm from the phallic edge connected to a convex curve taken from the scrotal edge of the outstretched penoscrotal web. The web may be dropped or excised to recreate a new penoscrotal angle [43,45,46].

A systematic review of 11 articles on scrotal laxity and penoscrotal webbing found that Z plasty and V-Y plasty were commonly performed procedures, and the authors described their preferred aesthetic scrotoplasty which included a vertical resection of the excess scrotal skin along the ventral median raphe and a penoscrotal junction Z plasty [47].

Scrotal septum detachment in men undergoing plication for Peyronie’s disease resulted in a perceived increased penile length (87.5% vs. no detachment, 77.3%) [48]. Miranda-Sousa et al. evaluated whether the release of the penoscrotal web would optimize patient perception and satisfaction regarding penile length after penile implant surgery. At 3 months, an increase in penile length was reported in 83.7% of patients who had a release of the penoscrotal web vs. 2.7% who did not have the release. In addition, penile shortening was reported in 4.7% of patients who had a release of the penoscrotal web vs. 83.8% who did not have the release [46].

#### Suspensory Ligament Release

The suspensory ligament supports and stabilizes the penis. Detachment of the ligament from the pubic symphysis enables the penis to move forward [9]. A study of 42 patients requesting penile lengthening by division of the penile suspensory ligament from a variety of aetiologies, including penile dysmorphic disorder (n = 26) and Peyronie’s disease (n = 7), found a mean increase in stretch penile length by 1.3 ± 0.9 cm [49]. The authors found that the outcome was superior when the procedure was combined with the insertion of a silicone buffer (testicular prosthesis).

Zhang et al. performed a suprapubic liposuction, penile suspensory ligament release, and insertion of a folded acellular dermal matrix between the corpora cavernosa and pubis symphysis in 15 men with a buried penis. At 3 months, the mean increase in penile length was 2.4 ± 0.8 cm. The postoperative complications included edema, ecchymosis, and poor wound healing. All patients were satisfied with the final appearance [50].

A study of 303 penile implants showed that the release of the suspensory ligament during an infrapubic insertion of an inflatable penile prosthesis may maintain or even increase penile length [51].

### 3.5. Techniques for Increasing Corporal Penile Length

More invasive surgical techniques up to total phalloplasty can be used to increase the effective length and width of the penis. These methods should be recommended in the first instance to patients with true micropenis for whom the methods previously considered could be ineffective.

The most widely used technique is the one where the suspensory ligament of the penis is incised to release the penis from the pubis. It can be combined with plasty of the penopubic angle using the inverted “V-Y” technique and lipoplasty, but does not increase the length of the erect penis. In a study by Bin et al., patients with true micropenis or dysmorphophobia had a combined “V-Y” plasty incision, division of suspensory ligaments, and implantation of autologous saphenous vein or ePTFE vessel patches at tunica albuginea which was incised laterally for a length of 10 mm to expose cavernous sinusoid space. All patients had normal erectile function after the procedure, an increase in penile length of 2–5 cm in both erect and flaccid status, and an increase in girth of 1–3 cm in both erect and flaccid status during 3–5 years of follow-up, while only minor complications such as preputial oedema and stich granuloma were described [52].

#### 3.5.1. Penile Disassembly

To increase the actual penile length, Perovic et al. described the penile disassembly technique, during which the penis is separated into the neurovascular bundle (NVB) with glans, corpora cavernosa, and urethra [19]. Subsequently, a space is created between the glans cap and tips of corpora cavernosa and an autologous cartilage is inserted on the dissected tip of corpora. The penile parts are assembled, and the procedure can also be combined with ligamentolysis and plasty of the penopubic angle. During a follow-up of 3.3 years, authors reported a 2–3 cm increase in 13 patients and a 3–4 cm increase in 6 patients in both the flaccid and erect status. There was no evidence of erosion or infection of the cartilage used and 100% had normal erectile function, while 26.3% developed penile curvature which was treated conservatively with penile stretch and vacuum devices [19].

#### 3.5.2. Total Phalloplasty

Phalloplasty is a complex reconstructive technique, commonly utilized for transgender patients, but can also be used in patients with penile length loss after trauma, penile cancer surgery, amputation, congenital micropenis, or reconstructive surgeries for epispadias/bladder exstrophy and hypospadias. In general, two flaps are created from a body area and are used to form a neophallus around a neourethra that is formed (“tube within a tube” technique). These flaps can be harvested from several body areas including the scapula, radial forearm, buttock, and latissimus dorsi, but the most commonly used for good cosmetic results is the radial forearm flap. The neourethra can be either anastomosed with the native urethra, if present; otherwise, it can be temporarily drained as a perineal/scrotal urethra, and a subsequent urethroplasty can follow at a second stage. A neo glans can be formed usually some months after the formation of neophallus/neourethra, and the final third stage is usually the insertion of a penile prosthesis. Functional and cosmetic outcomes are satisfactory in most patients, with flap survival of 96–100% in most series, patient satisfaction of 80–100%, urination in the standing position 50–100%, ejaculation 76–100%, and neophallus sensation of varying levels in almost all patients [20,21,22,23,24,25,26,27,28,29,53]. Complications can be minor with hematoma and penile oedema which most commonly resolve with conservative treatment. However, the most common type of complication is from urethral anastomosis, such as stricture (6.5–38%) and urethral fistula (12.5–50%) [20,21,22,23,24,25,26,27,28,29,53]. Necrosis of the flap is rare at 3.7–6.7%, and infection of the prosthesis with the need for removal ranges between 6.5 and 20% [20,21,22,23,24,25,26,27,28,29,53].

#### 3.5.3. Sliding Elongation

A newer technique, initially described by Rolle et al. in 2012, is the sliding elongation, during which several incisions of tunica albuginea are performed and, after a sliding between the parts created in the corpora cavernosa from the tunica incisions, the penile length is increased [31]. The penile length gain occurs in both flaccid and erect status and is mainly dependent on the flexibility of the NVB and urethra. The defects that were created by the incisions in tunica albuginea are covered with grafts, such as porcine pericardium or Tachosil, and in most cases a penile prosthesis is inserted [31]. The technique is utilized in most studies for patients with severe erectile dysfunction, Peyronie’s disease, and short length either due to curvature or after treating prostate cancer (radical prostatectomy or combination of hormonal therapy and radiotherapy) [30,31,32,33]. Several modifications of this technique have been described regarding either the site/shape and size of tunica albuginea incisions or the closure of defects with Buck’s fascia instead of graft material [34,54,55] (techniques described in detail in Table 1). Reported functional results are encouraging with an increase in length of 3.1–4 cm, increased girth of 1.6 cm, sensation in almost all patients with reported temporary penile numbness in 3–5%, and permanent loss of glans sensation in one patient in one study. Correction of curvature was observed in all patients, no erectile dysfunction was reported, and satisfaction of patients ranged between 90 and 100% [30,31,32,33,34,54,55]. In all studies, a significant increase in IIEF scores was noted during follow-up. Complications were mostly minor with hematomas in up to 25%, transfusion in 3.5%, infection and removal of the prosthesis in 0–7%, and glans necrosis in 0.7% [28,29,30,31,32,33,34].

### 3.6. Techniques for Increasing Penile Girth

Penile girth enhancement has been a subject of increasing interest and significance in the field of urology and sexual medicine. Men seeking to improve their sexual confidence or address concerns related to penile size often explore various techniques aimed at increasing penile girth. While penile length has traditionally garnered more attention, recent advancements have led to the development of multiple techniques specifically targeting girth enhancement.

Techniques include soft tissue fillers, grafting procedures, biodegradable scaffolds, and penile implants. Each technique is evaluated based on its clinical outcomes, durability of results, and complications.

#### 3.6.1. Soft Tissue Fillers

The first reported technique was penile autologous fat injection. The fat suctioned with a liposuction apparatus is divided into syringes after being filtered and is then injected into the penile tissue with the assistance of a cannula, thus ensuring equal distribution. Significant improvement in both the IIEF-5 score and intercourse satisfaction score were recorded along with a 32.2% increase in penile circumference. No adverse reactions or need for a second surgery were reported, except for one case of nodular fat occurrence [54].

Casavantes et al. described Polymethylmethacrylate (PMMA) microsphere injections to enhance penile girth in 729 cases of men with penile girth dissatisfaction. A significant mean increase of girth of 2.4 cm for the mid-shaft of the flaccid penis was recorded, even though 52% of men experienced many irregularities and 0.4% of them required a PMMA nodule removal [55].

Restylane Sub-Q (Q-med, Uppsala, Sweden) is a hyaluronic acid (HA) gel used in 41 cases to enhance penile girth. It was injected into the subcutaneous tissue of the penile shaft in a linear threading technique using a small needle. The gel was evenly distributed with multiple passes, also to achieve the desired girth increase according to patients’ needs and goals. Significant increases in penile girth both in flaccid and erect states at 18 months were recorded, along with improved patient self-esteem and satisfaction in the absence of major complications [56].

In a prospective multicenter double-blind randomized trial, HA was compared to polylactic acid (PLA) in 74 patients. During the injection, the needle was indwelled at the penile base at 1–2 and 10–11 o’clock positions with a volume range between 10 and 22 mL. At 18 months, the mean penile girths had significantly increased in both groups and satisfaction levels were significantly higher than those at baseline in both cohorts. Injection-associated adverse events (AEs) occurred in three (9.1%) patients in the HA group and in two (5.9%) patients in the PLA group, with no serious AE reported [35].

#### 3.6.2. Grafting Procedures

Different grafting materials and techniques have been reported to enhance penile girth.

Austoni et al. reported an augmentation phalloplasty with bilateral saphena grafts in a case series of 39 patients with either hypoplasia of the penis or functional penile dysmorphophobia. After penile degloving, a bilateral longitudinal incision was made in Buck’s fasci, a bilateral longitudinal incision was made in the albuginea cavernosa, and a venous graft capable of filling the opening was prepared, isolating and removing the saphena from its attachment to the femoral vein. The flaps obtained were shaped with a scalpel, to perfectly fit the shapes of the openings made in the albuginea, and then sutured. No major complications and specifically no losses of sensitivity of the penis or erection deficiencies occurred during the postoperative follow-up period and all the patients resumed their sexual activity in 4 months. The average penis diameter during erection was found to be 4.2 cm (3.4–4.9) with post-surgery increases in diameter varying from 1.1 to 2.1 cm (*p* < 0.01) [57].

In a single case report, a penile girth augmentation using flaps was performed, known as Shaeer’s augmentation phalloplasty. The superficial circumflex iliac artery island flap was used to increase penile girth for the first time. The superficial circumflex iliac vessels were identified, and the groin flap was elevated from lateral to medial, rotated toward the penis, and tunneled into a penopubic incision. It was wrapped around the penis short of the corpus spongiosum and insinuated under the glans. Six months after surgery, the patient had an erect girth of 19.5 cm and a flaccid girth of 16.5 cm, compared with 11 cm and 7 cm, respectively, before surgery, thus maintaining the intraoperative girth gain. Edema and congestion of the penis and scrotum were observed postoperatively, along with an area of sloughing on the dorsum of the penis, which re-epithelialized spontaneously [58].

Penile girth augmentation was also performed using a porcine dermal inteXen graft in a case series of 39 men with penile dysmorphophobia. A 3- to 4-cm-long incision was made along the penopubic junction through Colles’ fascia and extended to Buck’s fascia, which was preserved. The penis shaft was degloved from its skin and the dermal graft was tailored according to the desired shape and size and applied to the degloved penis shaft from the coronal sulcus to the base. The xenograft was placed circumferentially, dorsally, from the groove between the cavernous and spongious bodies from one side to the other and then sutured. Augmentation of 40% and 22% of the girth of the penis was reported in flaccidity and erection, respectively, along with improved sexual self-esteem and patient satisfaction. No major complications occurred in the series. Minor complications, including seroma, lumps, ecchymosis, and suture dehiscence, were resolved with conservative treatment within 3 weeks [59].

Girth augmentation of the penis using the Superficial Circumflex Iliac Artery and Vein (SCIAV) flap was reported in 52 patients. After being mobilized from the groin, the flap was tunneled under the pubic region to emerge at the base of the penis and then sutured to the subcoronal area and on either side of the spongiosum. An increase in flaccid girth from 9.3 cm to 14.5 cm was observed with additional improvement in flaccid non-stretched visible length. Re-surgery was needed for either de-bulking of the oversized flap, flap pedicle, or for donor site scar revision, while edema (resolved in 2–8 weeks) and dorsal shaft skin ulceration in overweight participants were also reported [59].

Acellular Dermal Matrix (ADM) has also been employed to improve penile girth, after being wrapped around the degloved penile shaft. At the 3-month follow-up, the penile circumference was increased by 1.1 cm on average. The overall complication rate was 71.8%, including 47 patients with erectile discomfort, 12 with delayed healing, 10 with unobvious augmentation effect, 8 with wound hematoma, 7 with prepuce edema, 4 with wound infection, and 3 patients with skin necrosis of the dorsal side. Seven patients eventually underwent ADM removal [60].

Recently, Adhikari reported outcomes of ten patients operated on for penile girth augmentation using dermofat grafts and SEPA (superior external pudendal artery) flaps. At 6-month follow-up, the final girth increase varied from 1.9 to 2.6 cm, and complications were described in up to 50% of patients including skin loss, urinary obstruction, and fat necrosis [61]. 

#### 3.6.3. Biodegradable Scaffolds

The dry polylactic-co-glycolic acid (PLGA) scaffold, a copolymer composed of lactic acid and glycolic acid, in association with autologous fibroblasts has been used to increase penile girth. Fibroblast cells harvested from biopsied scrotal dermal tissue are expanded in culture and suspended cells in culture medium are then seeded on pretreated tube-shaped PLGA scaffolds and incubated for 24 h. The scaffolds are then transplanted between Dartos and Buck’s fascia or under the neurovascular bundle. Girth improvement is reported between 2 and 3 cm, with positive ratings from patients. Postoperative complications may occur as an infection, penile skin pressure necrosis, or seroma formation, usually treated conservatively [62,63,64].

#### 3.6.4. Subcutaneous Penile Implant: “Penuma^®^”

A recently developed penile implant made of silicone is a Penuma^®^ implant and can be inserted to increase penile length in flaccid status [37,65]. This is a subdermal implant inserted through a transverse incision above the symphysis pubis or scrotum and sutured below the glans and at the base of the penis [37,65]. The reported mean increase in penile length was 4.9 cm in one study and 2.1 cm in another, while the increase in girth ranged between 39.9 and 56.7% [37,39,65]. The reported removal rate during follow-up of up to 4 years is 3–10%, while rest complications are usually minor [37,65]. Penuma^®^ has been recently cleared by the FDA for aesthetic enhancement of the flaccid penis [38]. 

## 4. Discussion

Penile augmentation surgeries have garnered attention throughout history, stemming from ancient practices to modern medical advancements. These procedures aim to address concerns regarding penile size, impacting men’s sexual confidence and satisfaction. The rising demand for such surgeries reflects the societal emphasis on physical appearance and sexual performance.

Whatever the reason and/or the medical condition driving patients to seek penile augmentation surgeries, the decision to undergo penile enhancement surgery necessitates careful consideration. Psychological assessments are pivotal, particularly in distinguishing between individuals with genuine concerns and those experiencing small penis anxiety. Proper diagnosis, involving comprehensive clinical evaluations, hormonal profiling, and psychiatric assessments, helps determine whether patients would benefit from medical therapy or invasive procedures [66].

Concerning surgical approaches, they target both penile length and girth enhancement. Techniques such as lipoplasty, skin reconstruction plasty, and suspensory ligament release primarily aim to improve perceived length [17,41]. These procedures have shown promising outcomes, albeit with certain limitations, such as postoperative complications and varying levels of patient satisfaction [17].

Conversely, more invasive methods including penile disassembly [19] and total phalloplasty offer substantive length gains [20]. While these procedures cater to patients with true micropenis, they pose higher risks and complexities, often necessitating multiple stages for reconstruction and potential complications involving urethral anastomosis and flap survival [20,21,22,23,24,25,26,27,28,29,53].

Girth enhancement techniques, including soft tissue fillers, grafting procedures, biodegradable scaffolds, and subcutaneous penile implants, have gained traction. These procedures exhibit a spectrum of outcomes and complications, with some showing promising results in increasing penile girth. However, complications such as infection, hematoma, and dissatisfaction have been reported across different techniques, highlighting the need for cautious consideration.

The majority of studies conducted in the past decade examining penile enhancement procedures in both healthy men and those with concurrent penile disorders have reported successful increases in penile dimensions or corrections of deformities, with only a few significant complications [31]. However, it is important to note that the scientific evidence relies heavily on studies with inadequate internal validity, such as observational designs, non-standardized methodologies, and heterogeneous populations.

The papers analyzed in this review demonstrated inconsistent approaches in evaluating changes in penile dimensions, highlighting the absence of a consensus in assessing and reporting efficacy outcomes. Previous reviews have also acknowledged the lack of valid methods for evaluating outcomes, particularly in procedures for aesthetic purposes [10,67,68]. The strength of this review lies in a comprehensive analysis of interventions performed for both aesthetic and therapeutic reasons in patients with concurrent penile disorders. Additionally, it does not exclusively focus on a specific group of interventions (surgical or non-invasive) or a particular condition or disease (e.g., PD), providing a comprehensive picture of the current landscape. Lastly, it examines interventions aimed at enhancing both the length and circumference of the penis.

All things considered, future advancements and research should focus on different areas to solve the following unmet needs.

Longitudinal comparative studies with standardized reporting: Conducting large-scale, long-term comparative studies between surgical methods and non-invasive approaches with comprehensive cohorts can provide more robust data on the effectiveness, safety, and longevity of different techniques. These studies should also be conducted following standardized reporting criteria for surgical outcomes and complications, using uniform data collection and analysis, facilitating better comparisons.

Technological innovation: Continuous refinement of surgical techniques and improvements in grafting methods, more advanced soft tissue fillers, or biodegradable scaffold designs can enhance safety and effectiveness. Furthermore, advancements in biomedical engineering might lead to the development of novel biomaterials or implants specifically tailored for penile augmentation, aiming for improved biocompatibility and durability. Lastly, the improvement of advanced imaging modalities or simulations can assist surgeons in preoperative planning and predicting surgical outcomes.

Patient care and education: Preoperative counseling to manage patient expectations and provide a realistic understanding of outcomes should be enhanced and potential complications should be managed by following standardized postoperative care protocols to minimize complications and improve recovery rates. This approach should be incorporated with psychological support, including counseling or therapy, to address body dysmorphic disorders or psychological distress related to genital size concerns.

Ethical consideration and education: Stricter regulations or guidelines in the field of penile augmentation surgery to ensure patient safety, adequate training of surgeons, and ethical practice should be developed.

Collaboration and interdisciplinary approaches: Collaboration between urologists, psychologists, sex therapists, and plastic surgeons should be encouraged to adopt a comprehensive approach to patient assessment, treatment planning, and postoperative care. This collaboration should also be encouraged between different institutions to facilitate data-sharing and to enhance understanding of patient outcomes.

In conclusion, while penile augmentation surgeries offer potential solutions for individuals concerned about genital size, they involve considerable risks and complexities. Rigorous research, standardized protocols, and advancements in surgical techniques are imperative to ensure optimal outcomes and patient satisfaction.

## 5. Conclusions

The review showed the wide landscape of penile augmentation surgeries, highlighting both the surgical techniques and the psychological considerations pivotal to patient selection. The limitations of the current research underscore the need for more robust studies to guide clinical practice and enhance patient outcomes.

## Figures and Tables

**Table 1:** Characteristics of studies on techniques for increased penile length.

Author/ Year	Study Design	N	Diagnosis (n)	Follow-Up	Technique	Outcomes	Complications
LIGAMENTOLYSIS-PLASTY OF PENOPUBIC ANGLE-INTERPOSITION OF MATERIAL IN TUNICA ALBUGINEA							
Benson 2020 [18]	Prospective case series	20	Micropenis (17) and dysmorphophobia (3)	5 years	- ‘’V-Y’’ plasty incision in anterior pubic symphysis - Division of suspensory ligaments - Incision (10 mm) of tunica albuginea - Implantation of either saphenous vein grafts or ePTFE vessel patches between lateral margins of incised tunica albuginea	- 100% (20) normal erectile function - Increase of penile length 2–5 cm in both flaccid and erect states - Increase of penile girth 1–2 cm in flaccid and 1.5–3 cm in erect state - 0.5–1 cm decrease in penile length and girth in 1 year follow-up in some patients - Similar results during 3–5 years of follow-up - Normal shape	- 100% (20) preputial edema which resolved in 2–3 weeks and in 1 patient circumcision - 1 stich granuloma
PENILE DISASSEMBLY							
Perovic 2000 [19]	Prospective case series	19	Short penis for satisfactory sexual intercourse (<10 cm)	3.3 years	- Separation of the penis into glans cap with the neurovascular bundle (NVB), corpora cavernosa, and urethra - Creation of space between glans cap and tips of corpora cavernosa - Insertion of autologous cartilage from patient ribs and re-joining of dissected glans-corpora cavernosa - ± ligamentolysis and plasty of penopubic angle	- 2–3 cm increase in 13 patients and 3–4 cm increase in 6 patients - No evidence of erosion or infection/inflammation of cartilage - 100% (19) normal erectile function - Painless sexual function	- 26.3% (5) penile curvature, treated with penile stretch and vacuum devices (VD)
PHALLOPLASTY							
Callens 2013 [20]	Cross-sectional	10	Penile deficiency with stretched penile length < 6 cm	36.9 months	- Modified radial forearm free flap technique - Penile prosthesis insertion after 10–25 months	- 100% (10) flap survival - 100% (10) patients satisfied with surgical results - 50% (5) urinated via the urethra and 50% (5) via diversion stoma - Penile prosthesis in 90% (9) patients - Satisfaction for sexual activity 7.3/10 - 100% (10) experienced ejaculation	- 10% (1) pulmonary embolism - 10% (1) Hematuria with clots and retention - 50% (5) urethral fistula and 30% (3) urethral stenosis - 20% (2) patients required closure of fistula
Lumen 2008 [21]	Retrospective case series	7	Bladder exstrophy (3), correction of hypospadias (1), infected foreign body (1), and penile amputation (2)	20 months	- 6 patients had radial forearm flap - 1 patient anterolateral thigh flap	- 100% flap survival - 4 patients pass urine through the urethra - 2 patients required closure of fistula - 1 patient required urethroplasty - 6 patients reported sensation in the neophallus - 6 patients reported high satisfaction and 1 moderate - 4 patients had prosthesis insertion, 2 of which were removed due to infection	- 1 pulmonary embolism - 1 clot retention - 2 fistulas at the anastomosis of neourethra with the native urethra - 1 urethral stricture - 1 hypertrophic scar
Falcone 2020 [2]	Prospective cohort study	108	30% bladder exstrophy, 26% micropenis, 26% penile cancer, and 18% trauma	78.5 months	Radial artery forearm free flap phalloplasty in 3 stages with 6-month intervals: total phallic reconstruction, glans formation with urethroplasty if needed, and inflatable penile prosthesis implantation	- 83.4% (90) had primary anastomotic urethroplasty - 71% (77) completed all stages of reconstruction - 96.3% (104) flap survival - 80% (86) satisfied with results - 100% (108) had some degree of penile sensation (20%, 1/5; 15%, 3/5; 26%, 3/5; 24%, 4/5; 15%, 5/5) - 76% (82) reach orgasm - Multivariate regression showed that staged urethroplasty was the significant risk factor for urethral complications but no factor predictive of vascular complications	- 3.7% (4) acute arterial thrombosis and complete loss of phallus - 1.8% (2) venous thrombosis and flap congestion treated with revision of venous anastomosis - 15.7% (17) penile infection and 2.8% (3) abscess - 6.5% (7) penile hematoma - 2.8% (3) penile scarring - 49.1% (53) had urethral complications
Garaffa 2014 [22]	Retrospective case series	16	Epispadias-bladder exstrophy	20.5 months	Radial artery forearm free flap phalloplasty in 3 stages with 6-month intervals: total phallic reconstruction, glans formation with urethroplasty if needed, and inflatable penile prosthesis implantation	- 87.5% (14) good penile sensation - 93.8% (15) satisfied with cosmesis - 93.8% (15) able to urinate/ejaculate from meatus - 100% (12) can have intercourse after insertion of prosthesis	- 12.5% (2) acute thrombosis - 6.3% (1) distal necrosis requiring anterolateral thigh flap - 12.5% (2) fistula - 37.5% (6) stricture - 18.8% (3) revision of prosthesis
Massanyi 2013 [23]	Retrospective case series	10	Bladder exstrophy (8) and cloacal exstrophy (2)	14 months	Radial artery forearm free flap phalloplasty in 3 stages with 6-month intervals: total phallic reconstruction, glans formation with urethroplasty if needed, and inflatable penile prosthesis implantation	- 100% (10) adequate sensation - 100% (10) able to reach orgasm - 100% (10) satisfied with cosmesis	- 10% (1) partial necrosis - 10% (1) acute thrombosis - 10% (1) forearm neuroma - 10% (1) stricture - 20% (1/5) infection of the prosthesis - 40% (2/5) removal of the prosthesis due to skin erosion
Ricketts 2009 [24]	Retrospective case series	5	Bladder exstrophy	2–4 years	Not Applicable (NA)	- 60% (3) would undergo operation again - 80% (4) very satisfied with cosmetic results - 66.6% (2/3) highest score for sexual function	- 20% (1) anastomotic thrombosis - 20% (1) groin cellulitis
Ma 2011 [25]	Retrospective comparative study	45	Trauma	9.1 years	Radial forearm free flap placement (28 received an innervated and 17 a non-innervated flap)	- 86% (24) of innervated and 0% of non-innervated could sense vibration - 80% (22) of innervated and 50% (8) of non-innervated could reach orgasm - 75% (21) of innervated and 12% (2) of non-innervated could distinguish blunt from sharp prick at the distal part - 100% (28) of innervated and 88% (15) of non-innervated could distinguish blunt from sharp prick at the proximal part	NA
Garaffa 2009 [26]	Case series	15	Subtotal penectomy for penile cancer	18.2 months	Radial artery forearm flap with a full thickness skin graft from the abdominal area (4/15), buttock (11/15)	- 40% (6) completed all 3 stages, 40% (6) 2 stages, and 20% (3) one stage of the procedure - 100% (15) able to void standing - 80% (12) had sensation to phallus - 6.7% (1) had sensation to the neourethra, 6.7% (1) had no sensation to the phallus - 100% (15) were satisfied with cosmetic results and size - 86.7% (13) able to have intercourse	- 6.7% (1) recurrent bulbar tumor - 6.7% (1) penile implant explanted due to infection - 13.3% (2) partial skin necrosis - 13.3% (2) phallic contracture - 20% (3) meatal stricture - 6.7% (1) anastomotic stricture - 33.3% (5) urethral fistula - 13.3% (2) arm contracture - 13.3% (2) loss of arm sensation - 6.7% (1) hand edema
Perovic 2007 [27]	Retrospective case series	16	Congenital penile anomaly (12), iatrogenic (2), and trauma (2)	31 months	- Latissimus dorsi flap - Mobilization of free flap and formation of neophallus on site - Transfer to pubic region and anastomosis of vessels - (3 months after) Buccal mucosa urethroplasty	- 100% (16) flap survival	- 12.5% (2) urethrocutaneous fistula
Yang 2007 [28]	Retrospective case series	20	Penile amputation after accident (12) and self-amputation (8)	1–5 years	Use of scapula flap	- 100% (20) able to void while standing - 75% (15) satisfactory intercourse, 15% (3) partially satisfied, and 10% (2) dissatisfied	- 15% (3) urethral fistula
Wang 2007 [29]	Retrospective case series	15	Micropenis (2), infection (1), burn (8), and self-amputation (4)	0.5–5 years	Use of scapula flap	- 93.3% (14) satisfied with cosmetic and functional results	- 6.7% (1) loss of flap viability
SLIDING ELONGATION-TUNICA EXPANSION PROCEDURES							
Egydio 2013 [30]	Prospective cohort study	105	Severe Peyronie’s disease and erectile dysfunction (ED)	18.2 months	Sliding technique	- 1% (1) infection of penile prosthesis - 99% (104) able to complete intercourse - Mean functional penile length gain of 3.6 cm - 2.9% (3) developed residual curvature up to 30° but this was not limiting - 100% (105) had sensation of glans and achieved orgasm and ejaculation - 89.4% (93) were satisfied with cosmetic and functional results - 95.2% (99) were satisfied with penile length gain, 3.8% (4) somewhat satisfied, and 1% (1) dissatisfied despite penis being functional	- No intraoperative complication was noted
Rolle 2012 [31]	Prospective case series	3	Peyronie’s disease with penile shortening and ED	13 months	Sliding technique	- Increase in length was 4, 2.5, and 3 cm in the 3 patients - 100% (3) had satisfactory intercourse with no loss in sensation or sign of vascular distress - IIEF score raised from 24 to 44 at 3 months, 50 at 6 months, and 60 at 12 months	- No major intraor postoperative complication was noted
Egydio 2015 [32]	Prospective cohort study	143	Severe Peyronie’s (77), severe ED (30), radical prostatectomy (21), hormonal therapy with radiotherapy for prostate cancer (10), penile fracture (3), priapism (1), and hypospadias repair (1)	9.7 months	Sliding technique as proposed by Rolle but tunical defects were closed with Buck’s fascia instead of graft–Modified Sliding Technique (MOST)	- Mean penile length gain was 3.1 cm - 0% penile prosthesis infection - 100% (143) retained glans sensation and the ability to have intercourse - 100% (77) had corrected their penile curvature - IIEF score increased from 24 at baseline to 60 at 6 months of follow-up	- 24.5% (35) penile shaft hematomas which resolved spontaneously - 4.9% (7) temporary penile numbness
Rolle 2016 [33]	Prospective cohort study	28	Stable Peyronie’s disease	37 months	Sliding technique. Graft was porcine intestinal submucosa in 19 patients, Pelvicol in 2 patients, and Tachosil in 7 patients	- Malleable prosthesis in 3 patients and inflatable three-piece in 25 patients - 3.5% (1) reported permanent loss of glans sensation - Mean penile lengthening 3.2 cm - 100% success in the management of curvature - Mean IIEF score improved from 27 at baseline to 45 at 3 months, 57 in 6 months, and 64 in 12 months - 95% (27) satisfied with increase in penile length	- No intraoperative complications - 3.5% (1) blood transfusion - 7% (2) infection and removal of prosthesis - 56% (16) hematoma formation with conservative management
Egydio 2018 [34]	Prospective cohort study	138	Peyronie’s disease (83), severe and therapy-resistant ED (34), radical prostatectomy (14), androgen-deprivation therapy with or without brachytherapy or external beam radiotherapy for prostate cancer (5), and penile fracture (3)	15.2 months	Modified sliding technique (MUST-Multiple Slit Technique)	- 3-piece inflatable prosthesis in 35 patients and malleable prosthesis in 103 patients - 2.9% (4) temporary glans numbness - 2.2% (3) reported concerns regarding penile length and girth increase - Mean penile length gain of 3.1 cm - 5.1% (7) temporary anorgasmia - 100% (83) of patients with curvature had no residual curvature - 0% hypermobility of glans since glanspexy was performed when necessary - Mean IIEF score increased from 22 to 61 at 6-month follow-up	- 0.7% (1) glans necrosis - 18.8% (26) hematomas resolved spontaneously - 0% penile prosthesis infection
Egydio 2020 [35]	Prospective cohort study	416	Peyronie’s disease (287), severe and therapy-resistant ED (65), radical prostatectomy (50), androgen-deprivation therapy with or without radiotherapy (10), and penile fracture (4)	1 year	Modified tunica expansion procedure (TEP strategy) A penile prosthesis inserted at the end of the procedure and glanspexy was performed if needed	- 5% (21) reported concerns regarding small changes in length and girth - 100% correction of curvature - Mean penile length gain of 3.3 cm - Mean IIEF score increased from 22 at baseline to 68 at 6-month follow-up - 92.8% (386) had glanspexy	- 19.9% (83) hematoma conservatively managed - 3.8% (16) partial glans numbness - 7% (29) transient anorgasmia - 0% glans necrosis - 0.2% (1) penile prosthesis infection
Razdan 2022 [36]	Retrospective cohort study	32	Severe ED and Peyronie’s disease (24), radical prostatectomy (8)	1 year	Tunica expansion procedure (TEP) using a scrotal incision	- Mean increase in length 2.8 cm - Mean increase in girth 1.6 cm - Mean persistent curvature 5 degrees	- No reported complications
PENUMA SILICONE IMPLANT							
Elist 2018 [37]	Retrospective cohort study	400	Patients with a perception of small penis, buried penis from prepubic recession, micropenis	4 years	PENUMA silicone implant inserted either with the infrapubic or the scrotal incision	- Mean penile girth at midshaft increased by 56.7% from 8.5 cm to 13.4 cm - The size of the glans was unchanged - Mean penile flaccid length was 9.1 cm preoperatively and 11.3 postoperatively - Self-confidence increased from 2% to 91.5% after 6–8 weeks and this was maintained for 83.5% during 4 years of follow-up - 0% erectile dysfunction	- 4.8% (19) seroma requiring compressive pressure in 12 and aspiration in 7 - 4.5% (18) had hypertrophic scar and required therapy in 10 after 3–5 months - 3.2% (13) of wound infections between 5 and 12 months after the procedure, 5 of which were treated with oral antibiotics and 8 (2%) device was removed - 1.5% (6) temporary loss of glans sensitivity - 1% (4) hematoma - 1.5% (6) implant malposition - 3% (12) overall need for device removal (implant breakage, infection, suture detachment, and hematoma)
Wilson 2022 [38]	Retrospective cohort study	100	NA	NA	PENUMA silicone implant inserted through a scrotal incision	- High/very high satisfaction 57% (57) - Medium/high satisfaction 35% (35) - Medium/low satisfaction 2% (2) - Low/very low satisfaction 6% (6)	- 10% (10) had the implant removed, 4 due to infection, 3 due to suture dehiscence, 1 due to both infection and suture dehiscence, and 2 due to desire to remove it
Siegal 2023 [39]	Retrospective cohort study	49	Patients with a perception of a small penis, buried penis from prepubic recession, or micropenis	6 months	PENUMA silicone implant inserted either with the infrapubic or the scrotal incision	- Length increased by 52% and a mean of 4.9 cm - Increase in girth by 39.9% - 0% ED	- 2% (1) infection - 4% (2) erosion - 6.1% (3) revision surgery due to persistent flaring of the implant - 0% seroma

NVB = neurovascular bundle, VD = vacuum device, ED = erectile dysfunction.

## Data Availability

The authors confirm that the data supporting the findings of this study are available within the article.
